# Odor Mortis

**Published:** 1881-08

**Authors:** 


Odor Mortis.—Dr. A. B. Isham, speaks of a peculiar odor, in the *Am. Jonrn. of Med. Science* for April of this year, which will doubtless recall similar experiences of other physicians at the death-bed side. It is somewhat strange that an account or description of this phenomenon should have remained so long unnoticed, or at least so little noticed, in medical writings, as it has doubtless been observed in thousands of cases and from the earliest times. Dr. Isham describes the odor as moschifous, possessing an appreciable, yet indescribable difference from the odor of musk; the impression upon the olfactory organs being more delicate, more subtle. We often receive impressions at the bedside which possess us decidedly with the idea of approaching dissolution, without being able to satisfy our minds, when attempting an analysis of the phenomena presented by the patient, as to the special symptom or sign denoting death; and yet whilst the *tout ensemble* fixes the conviction in the mind of the observer, no single phenomenon would be able to do so in the particular case. In a brief retrospect of our experience at death-beds, we are made aware of the importance of recognising the *odor mortis* in forming a prognosis where it exists. We recall cases where it was distinguishably present, and whilst not duly estimated at the time, was an important factor in making up a decided judgment as to the fatal termination of the case. Dr. Isham has conferred a real advantage upon the profession in bringing the subject of this peculiar odor prominently before it. The fact that there are odors *peculiar* to certain diseases, is not, we apprehend, as mnch appreciated by the profession as they should be, and if so, our olfactories should be brought into requisition more frequently in the diagnosis and prognosis of diseases than at present.

Certain odors are *pathognomonic* of the diseases in which they occur. Thus small-pox, cholera and yellow fever have each an odor so peculiar in character that when learned or experienced by a physician, will enable him to make a correct diagnosis of either, when brought sufficiently near such a case, even in the dark. No one would be liable to err in a case of cancer uteri, who has ever smelt the odor of that deplorable malady; and some of *our* contemporaries of two or more decades just passed, would not require to see the effects of calomel upon the mouth of a patient, if the peculiar odor of ptyalism should greet his olfactories upon entering a room! All this goes to show that the physician should educate all the senses, as far as possible, and that the culture of that of smell is not the least of them. Did time permit, some personal experiences, in regard to odors, in the diseases mentioned, and in others also, might be related, which would be found interesting to some of our readers; but we must defer them to some other occasion.

